# From Theory to Practice: Which Biomarkers Are Ready for Predicting Response in Advanced HCC?

**DOI:** 10.1111/liv.70684

**Published:** 2026-05-13

**Authors:** Marie Decraecker, Jean‐Frédéric Blanc, Samuel Amintas, Grégoire Manaud

**Affiliations:** ^1^ Univ. Bordeaux, Inserm UMR1312 BoRdeaux Institute of onCology (BRIC) Bordeaux France; ^2^ Department of Oncology Bordeaux University Hospital Bordeaux France

## Abstract

Systemic therapies for advanced hepatocellular carcinoma (HCC) have expanded considerably with the advent of tyrosine kinase inhibitors, immune checkpoint inhibitors and immunotherapy–anti‐angiogenic combinations. However, despite this therapeutic diversification, first‐line treatment selection remains largely empirical, as few biomarkers are available at diagnosis to inform therapeutic choice. This review provides a practice‐oriented synthesis of predictive biomarkers evaluated in advanced HCC, distinguishing those that are clinically transposable from those that remain exploratory. Among currently available tools, only a limited set of biomarkers demonstrates sufficient robustness and feasibility for real‐world use, and most provide information after treatment initiation rather than guiding upfront selection. Liver function assessment (ALBI score), dynamic Alpha‐fetoprotein (AFP) kinetics and anti‐drug antibodies (ADA) emerge as the most actionable parameters. ALBI retains predominantly prognostic value, whereas early AFP decline and ADA formation offer treatment‐specific, on‐treatment insights that may support clinical decision‐making. Imaging biomarkers such as mRECIST remain essential for early efficacy assessment but lack predictive value at baseline. Routine histological markers—including PD‐L1, mismatch repair proteins and β‐catenin immunostaining—do not reliably predict response prior to therapy initiation. In contrast, a broad range of circulating, tissue‐based and imaging‐derived biomarkers—including liquid biopsy analytes (cytokines, CTCs, ctDNA, miRNAs), transcriptomic immune signatures, artificial intelligence AI‐assisted histopathology, radiomics and multi‐omic tumour profiling—provide substantial mechanistic insight but remain investigational. Their limited clinical applicability reflects methodological heterogeneity, insufficient standardisation and the absence of prospective validation in biomarker‐driven trials. Among emerging approaches, proteomics stands out as a particularly promising strategy. By directly capturing the protein expression landscape of the tumour and its microenvironment, proteomics may overcome the inferential limitations of genomic and transcriptomic biomarkers and contribute to the development of biologically grounded, pre‐treatment stratification tools in advanced HCC. Ultimately, this review underscores a critical unmet need: the development of integrated, multi‐omic predictive strategies that combine baseline tissue characteristics with dynamic liquid biopsy markers and advanced imaging. Such approaches, strengthened by AI and prospective biomarker‐driven trials, are essential to move beyond empirical therapy selection and toward true precision medicine in advanced HCC.

## Key Points

Treatment options for advanced liver cancer have increased significantly in recent years, but choosing the best therapy for each patient is still largely based on clinical judgement rather than tumour biology. Although many potential biomarkers have been identified, none are currently used in routine care to guide treatment decisions. Distinguishing between biomarkers that can be used in everyday practice and those that remain experimental is essential to move toward more personalised treatment strategies. Systemic therapies for advanced hepatocellular carcinoma have expanded dramatically, yet treatment selection remains empirical. Although numerous predictive biomarkers have been proposed, none are currently integrated into clinical guidelines. Distinguishing clinically transposable biomarkers from exploratory ones is now essential to implement precision medicine in advanced HCC.

## Lay Summary

Systemic therapies for advanced hepatocellular carcinoma have expanded dramatically, yet treatment selection remains empirical. Although numerous predictive biomarkers have been proposed, none are currently integrated into clinical guidelines. Distinguishing clinically transposable biomarkers from exploratory ones is now essential to implement precision medicine in advanced HCC.

**Abbreviations**
A + B: atezolizumab plus bevacizumab
ABRS: atezolizumab–bevacizumab response signature
ADA: anti‐drug antibodies
AFP: alpha‐fetoprotein
AI: artificial intelligence
ALBI: albumin–bilirubin score
Ang‐2: angiopoietin‐2
CD: cluster of differentiation (e.g., CD3, CD8)
cfDNA: cell‐free DNA
CNA: copy number alteration
CR: complete response
CRAFITY: C‐reactive protein and AFP in immunotherapy score
CRP: C‐reactive protein
CTCs: circulating tumour cells
ctDNA: circulating tumour DNA
CTLA‐4: cytotoxic T‐lymphocyte–associated protein 4
DCR: disease control rate
DCs: dendritic cells
dMMR: deficient mismatch repair
EASL: European Association for the Study of the Liver
EV: extracellular vesicles
FFPE: formalin‐fixed, paraffin‐embedded
GO: gene ontology
H&E: haematoxylin and eosin
HCC: hepatocellular carcinoma
HGF: hepatocyte growth factor
HR: hazard ratio
ICI: immune checkpoint inhibitor
IFN‐γ: interferon gamma
IL: interleukin
LC–MS/MS: liquid chromatography–tandem mass spectrometry
LRP1B: low‐density lipoprotein receptor‐related protein 1B
MASH: metabolic dysfunction–associated steatohepatitis
MASLD: metabolic dysfunction–associated steatotic liver disease
miRNA: MicroRNA
ML: machine learning
mRECIST: Modified Response Evaluation Criteria in Solid Tumours
MS: mass spectrometry
MSI: microsatellite instability
NGS: next‐generation sequencing
NLR: neutrophil‐to‐lymphocyte ratio
NRP1: neuropilin‐1
ORR: objective response rate
OS: overall survival
PD: progressive disease
PD‐1: programmed cell death protein 1
PD‐L1: programmed death ligand 1
PFS: progression‐free survival
PLR: platelet‐to‐lymphocyte ratio
PR: partial response
PTM: post‐translational modification
RECIST 1.1: response evaluation criteria in solid tumours version 1.1
RNA‐seq: RNA sequencing
SD: stable disease
SII: systemic immune‐inflammation index
STRIDE: single Tremelimumab Regular Interval Durvalumab regimen
Teff: effector T cells
TILs: tumour‐infiltrating lymphocytes
TKI: tyrosine kinase inhibitor
TLS: tertiary lymphoid structures
TMB: tumour mutational burden
TME: tumour microenvironment
TP53: tumour protein p53
VAF: variant allele frequency
VEGF: vascular endothelial growth factor
VETC: vessels encapsulating tumour clusters

## 1 Introduction

Hepatocellular carcinoma (HCC) remains a major global health burden and is most often diagnosed at an advanced stage, when curative options are no longer feasible [1, 2]. Over the past decade, the systemic treatment landscape of advanced HCC has undergone profound changes, shifting from a sorafenib‐dominated era to a setting where tyrosine kinase inhibitors (TKI), immune checkpoint inhibitors (ICI) and ICI–anti‐angiogenic combinations coexist as first‐line options [3, 4, 5, 6, 7, 8]. These therapeutic advances have translated into improved survival for selected patients, yet the overall benefit remains highly variable, reflecting the profound biological heterogeneity of HCC and the frequent coexistence of underlying cirrhosis that impacts prognosis [9].

Despite major advances in systemic therapies, treatment selection in routine clinical practice remains largely empirical. Decisions rely predominantly on clinical parameters—liver function, performance status, comorbidities—rather than on the intrinsic biology of the tumour or its microenvironment. No validated biomarker currently guides the choice between immunotherapy‐based combinations and TKIs, and none are included in international guidelines [3, 10]. Importantly, most biomarkers currently available in clinical practice provide prognostic information or reflect treatment activity once therapy has started, rather than enabling true baseline treatment selection. In the context of systemic therapy, biomarkers can broadly be classified into three categories. (1) Predictive biomarkers identify patients more likely to benefit from a specific therapy before treatment initiation. (2) Prognostic biomarkers provide information on disease outcome independent of treatment choice, reflecting tumour aggressiveness or host factors. (3) Monitoring or on‐treatment biomarkers reflect treatment activity after therapy initiation and may help assess early response or emerging resistance. In advanced HCC, most currently available biomarkers belong to the latter two categories rather than enabling baseline treatment selection. As a consequence, a substantial proportion of patients experience early progression under systemic therapy, often accompanied by deterioration of liver function. This limits access to subsequent treatment lines and ultimately impacts overall survival. Recent real‐world evidence from large prospective cohorts has underscored this issue, showing that less than 25% of patients treated with ICI‐based combinations ultimately reach second‐line therapy [11, 12, 13, 14]. Interpretation of survival outcomes across studies should however be cautious, as overall survival in advanced HCC is strongly influenced by hepatic reserve, baseline liver function and access to subsequent treatment lines.

Meanwhile, numerous candidate biomarkers of response have been proposed in literature—ranging from simple serum markers to complex genomic, transcriptomic, radiomic and immunological signatures [15, 16, 17]. However, their level of validation, reproducibility and feasibility for routine clinical implementation vary considerably. Most remain exploratory, with limited standardisation, restricted availability or insufficient prospective evidence. This raises a critical question that has not yet been addressed in the literature: which biomarkers are truly ready for clinical use today, and which remain confined to translational research?

Several recent reviews have summarised the rapidly expanding landscape of molecular and immunological biomarkers in HCC [18]. However, most have primarily focused on biological mechanisms or exploratory translational approaches rather than on their clinical readiness or applicability in routine practice.

Addressing this question is essential to bridge the gap between research advances and clinical decision‐making. In this context, emerging technologies such as high‐resolution proteomic profiling offer new perspectives by capturing the functional state of tumour, its microenvironment and immune pathways beyond what genomics alone can reveal [19].

This review therefore proposes a critical, practice‐oriented appraisal of predictive biomarkers for systemic therapies in advanced HCC. Our aim is to identify biomarkers that are genuinely transposable to routine care—whether for baseline risk stratification, treatment monitoring or dynamic assessment of therapeutic response—while also highlighting promising but still immature approaches. We further discuss how innovative tools, including proteomics and anti‐drug antibody (ADA) monitoring, could contribute to the emergence of a pragmatic form of precision medicine in advanced HCC. To facilitate this interpretation, we also propose a conceptual framework classifying biomarkers according to their technical maturity and predictive relevance, distinguishing exploratory biomarkers from those with potential near‐term clinical transposability.

## 2 Methods

This review is a narrative, practice‐oriented synthesis of the literature on biomarkers associated with response to systemic therapies in advanced HCC. A literature search was conducted in PubMed and Web of Science to identify relevant studies published between January 2015 and March 2026 using combinations of the following keywords: ‘hepatocellular carcinoma’, ‘biomarker’, ‘immunotherapy’, ‘immune checkpoint inhibitor’, ‘atezolizumab’, ‘bevacizumab’, ‘tyrosine kinase inhibitor’, ‘liquid biopsy’, ‘tumour microenvironment’ and ‘proteomics’. Priority was given to prospective studies, translational analyses embedded in clinical trials and large retrospective cohorts investigating biomarkers associated with treatment response. Additional relevant articles were identified through reference lists of key publications and recent review articles. Studies were qualitatively assessed for their biological rationale, level of clinical evidence and potential transposability to routine clinical practice. For each biomarker, the level of supporting evidence was interpreted according to the degree of clinical validation available, distinguishing findings derived from preclinical studies, retrospective single‐centre analyses, multicentre retrospective cohorts, prospective studies and post hoc analyses of phase II/III clinical trials when available.

## 3 Clinically Applicable Biomarkers

The search for predictive biomarkers of response in advanced HCC has yielded a vast landscape of exploratory candidates, yet very few have crossed the threshold into real‐world clinical utility. Only biomarkers that are robust, reproducible, inexpensive and widely accessible can meaningfully guide treatment decisions today. This section highlights those limited but truly transposable tools.

### 3.1 Clinical Biomarkers

Clinical parameters continue to serve as the most readily available ‘biomarkers’ in advanced HCC, although they predominantly predict prognosis and treatment tolerance rather than treatment‐specific benefit. Within this category, liver disease aetiology and objective liver function metrics—especially Albumin–bilirubin score (ALBI)—have emerged as key modifiers of outcomes in the immunotherapy era. In routine practice, treatment selection is therefore often driven less by positive predictive markers than by the presence of contraindications to specific regimens, with comorbidities, bleeding risk, cardiovascular status and liver reserve frequently leading to a default rather than biologically guided choice.

#### 3.1.1 Liver Disease Aetiology

The potential impact of liver disease aetiology on the efficacy of immunotherapy has been intensely debated over the last few years. Experimental and translational data first raised concerns that non‐viral, and particularly metabolic dysfunction–associated steatohepatitis (MASH)‐related, HCC might be intrinsically less responsive to ICI. In the landmark Nature paper by Pfister et al. [20] NASH‐driven HCC in mouse models displayed impaired anti‐tumour immune surveillance, with dysfunctional CD8^+^ T‐cell responses and an exhausted, tissue‐damaging immune milieu, leading the authors to suggest that NASH–HCC could derive less benefit from immunotherapy. These findings, together with exploratory subgroup signals from early clinical trials, fuelled the hypothesis that non‐viral HCC—especially MASLD/MASH—might constitute a ‘poor‐responder’ population to ICI.

However, subsequent clinical data have been far more nuanced. Several real‐world cohorts and post hoc analyses have failed to confirm a clinically meaningful, consistent negative impact of non‐viral etiologies on outcomes with ICI‐based regimens. Pinto et al. [21] systematically reviewed the available evidence and concluded that, although early subgroup analyses suggested reduced ICI efficacy in non‐viral HCC, these data were not derived from trials stratified by aetiology and should be interpreted with caution. Roth et al. [22] similarly highlighted that, while aetiology may modulate tumour immunobiology, it does not currently justify withholding immunotherapy in non‐viral HCC in the absence of robust, prospective evidence.

More recently, large real‐world studies specifically addressing atezolizumab–bevacizumab (A + B) have shown remarkably consistent efficacy across etiological subgroups. These cohorts were largely composed of patients with preserved liver function (predominantly Child–Pugh A) treated in first‐line systemic settings. In the multicenter study by Rossari et al. [23] A + B demonstrated similar response rates and survival outcomes in viral versus non‐viral HCC, with overlapping toxicity profiles and access to second‐line therapies, leading the authors to conclude that A + B efficacy does not vary according to underlying aetiology. Other cohorts and post hoc analyses of the IMbrave150 trial corroborate this absence of major interaction between aetiology and A + B benefit [24].

At this stage, aetiology main role remains more descriptive rather than decisional in routine practice, and should not be used to deny ICI‐based combinations to patients with non‐viral or NASH‐related HCC.

#### 3.1.2 Baseline Liver Function

In contrast, liver function scores clearly fulfil the criteria of ‘transposable’ biomarkers: they are simple, reproducible, inexpensive and already used daily in decision‐making. Among these, the Albumin–Bilirubin (ALBI) grade has emerged as a particularly attractive tool [25].

The ALBI score, based solely on serum albumin and bilirubin, provides an objective and continuous assessment of liver functional reserve and has repeatedly been shown to stratify prognosis across stages and treatment modalities in HCC [26]. Compared with Child–Pugh, ALBI avoids subjective components (ascites, encephalopathy) and offers finer discrimination within Child–Pugh A patients, which is especially relevant in advanced HCC candidates for systemic therapy [27].

The ALBI score has emerged as one of the most robust and clinically transposable biomarkers in advanced HCC, but its role is essentially prognostic rather than truly predictive of treatment‐specific response. Importantly, the ALBI score primarily reflects hepatic functional reserve and overall risk stratification rather than tumour biology or treatment‐specific sensitivity. As such, it should be interpreted as a prognostic modifier of treatment tolerance and outcomes rather than a predictive biomarker guiding regimen selection. Across multiple systemic regimens, patients with ALBI grade 1 consistently achieve longer OS and PFS than those with ALBI 2–3. In most studies, these survival endpoints were calculated from systemic treatment initiation.

In sorafenib‐treated cohorts, median OS drops from about 10–12 months in ALBI 1 group to 6–8 months in ALBI 2 and < 4 months in ALBI 3 patients. In a large contemporary real‐world series of 406 patients receiving systemic therapy (TKIs and/or ICIs), da Fonseca et al. [28, 29] reported median OS of 12.5, 8.4 and 3.8 months for ALBI 1, 2 and 3 patients, respectively. Additionally, ALBI score was independently associated with survival (HR 1.66) and outperforming Child–Pugh in prognostic discrimination. Dynamic ALBI deterioration within the first month of systemic therapy was also associated with a doubling of the risk of death.

Similar patterns are observed with newer agents: in IMbrave150, A + B provided a substantial OS and PFS benefit over sorafenib in ALBI 1 patients (HR for OS 0.50), whereas no clear OS advantage was seen in ALBI 2 patients, despite a modest PFS gain. Conversely, in the HIMALAYA trial, the STRIDE regimen improved survival vs. sorafenib irrespective of ALBI grade, although absolute OS remained almost twice as long in ALBI 1 compared with ALBI 2/3 [30].

Taken together, ALBI appears as a powerful, inexpensive and fully transposable biomarker that quantifies hepatic reserve and strongly conditions the magnitude and duration of benefit from systemic therapy from a more prognostic tool. Its main clinical utility lies in determining whether systemic therapy is reasonable, how aggressive it can safely be and how likely a patient is to reach subsequent lines, rather than in choosing one specific first‐line regimen over another.

### 3.2 Simple Serum Biomarkers

Circulating serum biomarkers are among the most appealing candidates in advanced HCC due to their accessibility, low cost and routine use in clinical practice. Yet despite these advantages, most serum markers primarily provide prognostic information and offer limited ability to predict treatment‐specific benefit. Only a small number show genuine promise as early, transposable indicators of therapeutic response, underscoring the need to distinguish clinically meaningful tools from purely exploratory signals.

#### 3.2.1 Alpha‐Fetoprotein (AFP)

AFP is the most established serum marker in HCC, but its value as a baseline predictive biomarker remains modest. As highlighted in recent reviews, baseline AFP primarily retains prognostic value, whereas early AFP kinetics represents a dynamic on‐treatment biomarker reflecting treatment activity. Consistently across recent reviews and a large 2025 meta‐analysis—including 131 studies, predominantly retrospective cohorts of advanced HCC patients receiving immune checkpoint inhibitors, with survival outcomes generally defined from treatment initiation—baseline AFP behaves as a prognostic marker—reflecting tumour burden and biological aggressiveness—rather than a true predictor of treatment‐specific benefit. High baseline AFP was associated with worse OS (HR 1.60), PFS (HR 1.35), and lower disease control rates, yet showed no association with objective response to ICI therapy, confirming that AFP at treatment initiation cannot be considered a clinically transposable predictive biomarker [31].

In contrast, AFP becomes clinically meaningful through its early dynamic evolution [32]. Multiple studies—and confirmed in the 2025 meta‐analysis—demonstrate that early AFP decline has been consistently associated with improved treatment outcomes and may serve as a dynamic on‐treatment marker reflecting therapeutic activity [31]. A ≥ 75% reduction within the first 4–6 weeks under ICI‐based therapy has been consistently associated with higher radiological response rates and improved survival, while the meta‐analysis showed that even a > 20% decline significantly predicted superior OS (HR 0.41), PFS (HR 0.38) and markedly higher ORR (OR 5.39). Thus, dynamic AFP kinetics—not baseline AFP—represent the clinically actionable, on‐treatment biomarker with genuine predictive value in advanced HCC. Multiple studies have shown that a ≥ 75% decrease within 4–6 weeks of initiating an ICI‐based regimen strongly correlates with radiological response and survival.

#### 3.2.2 Inflammation‐Based Indices (NLR, PLR, SII)

Systemic inflammatory indices—neutrophil‐to‐lymphocyte ratio (NLR), platelet‐to‐lymphocyte ratio (PLR) and systemic immune‐inflammation index (SII)—have also been associated with outcomes. Elevated NLR or SII correlates with reduced survival under immunotherapy and TKIs in several retrospective series, likely reflecting an immunosuppressive myeloid‐driven systemic milieu [33]. However, their lack of standardised cut‐offs, susceptibility to confounding factors (infection, corticosteroids, cirrhosis‐related inflammation) and absence of prospective validation limit their applicability. These indices remain robust prognostic markers, but do not constitute predictive biomarkers in their current form. Nevertheless, given their universal availability and low cost, inflammation‐based indices represent attractive candidates for large‐scale prospective validation. Carefully designed studies will be required to define robust, reproducible thresholds and to clarify their potential role within composite, clinically actionable scores integrating host inflammation, liver function and treatment‐related variables.

Several recent studies have also explored the prognostic role of inflammatory indices specifically in patients treated with atezolizumab plus bevacizumab [34]. In a large international multicentre cohort, elevated baseline NLR (≥ 5) was associated with significantly worse overall survival, although no clear association with objective response was observed, supporting its role as a prognostic rather than predictive biomarker in this setting.

Composite scores have indeed attempted to enhance predictive accuracy. Among them, the CRAFITY score—combining AFP and C‐reactive protein—has demonstrated an ability to stratify patients according to their likelihood of deriving benefit from immunotherapy, including A + B [35]. While simple and reproducible, CRAFITY remains a probabilistic risk tool and cannot yet guide the selection of one systemic regimen over another.

#### 3.2.3 Anti‐Drug Antibodies (ADA)

In contrast, ADA—observed in post hoc analyses of prospective clinical trials‐ represent one of the few serum biomarkers with true potential for clinical transposability in advanced HCC. Immunogenicity against therapeutic monoclonal antibodies can reduce drug exposure and impair anti‐tumour activity. This mechanism—long recognised in immune‐mediated diseases—has recently gained attention in oncology. In IMbrave150, 29.6% of patients developed ADA against atezolizumab within 6 weeks. In HIMALAYA, ADA rates were lower but present for durvalumab (1.7%) and tremelimumab (4.4%) [36]. Their presence did not appear to impact clinical efficacy or safety of STRIDE or D monotherapy in the small number of ADA+ patients. A recent multicentre study published in JAMA Oncology reported that ADA can emerge as early as week 3 in 17.4% of patients treated with atezolizumab–bevacizumab, and were associated with lower response rates, reduced PFS and OS, and markedly decreased circulating atezolizumab concentrations [37].

These data indicate that ADA positivity is directly associated with early treatment resistance.

Importantly, ADA detection is feasible on standard ELISA‐based platforms, non‐invasive and reproducible—fulfilling key criteria for a clinically actionable biomarker. Unlike AFP or inflammation‐based indices, ADA provide treatment‐specific biological information, directly reflecting reduced drug bioavailability and efficiency.

### 3.3 Standard Imaging Biomarkers

Imaging remains a cornerstone of treatment monitoring in advanced HCC, and unlike many serum or tissue biomarkers, radiologic response assessment is already embedded in routine clinical workflows. Nevertheless, not all imaging criteria provide equivalent predictive value, and only a subset reliably captures the early biological effects of systemic therapies.

Radiologic evaluation remains essential for monitoring systemic therapy in advanced HCC, and among available tools, enhancement‐based criteria have proved more informative than traditional size‐based approaches. RECIST 1.1, although widely used, often fails to capture early biological effects of anti‐angiogenic therapies or immunotherapy, which may reduce tumour viability without immediate size reduction.

The modified RECIST (mRECIST) criteria were developed to address this limitation by quantifying only the arterially enhancing, viable portion of the tumour. This approach markedly increases objective response rates compared with RECIST 1.1 and correlates more strongly with OS [38, 39].

Recent analyses confirm that mRECIST is particularly effective for identifying true responders to anti‐angiogenic therapy and for detecting early treatment efficacy.

Other enhancement‐based methods, such as EASL and Choi criteria, have been explored but lack standardisation and have not achieved broad clinical adoption [40, 41, 42].

With the advent of immunotherapy, immune‐adapted criteria such as iRECIST were proposed to account for atypical patterns like pseudoprogression. However, pseudoprogression is rare in HCC, and major phase III trials have continued to rely on RECIST 1.1, sometimes supplemented by mRECIST. Comparative studies show that iRECIST adds little value over RECIST 1.1, whereas mRECIST more consistently identifies early clinical benefit [43, 44].

In practice, early mRECIST assessment at 6–8 weeks is one of the most robust indicators of treatment benefit and is already used routinely to guide decisions on therapy continuation or modification. Qualitative assessment of decreased arterial enhancement may suggest early response but remains non‐standardised and insufficiently reproducible to serve as a biomarker. Thus, mRECIST remains the most practical and clinically accepted enhancement‐based criterion.

## 4 Routine Histological Biomarkers

Histological evaluation remains a foundational component of cancer diagnostics, and immunohistochemistry (IHC) is routinely available in all pathology laboratories. However, in advanced HCC, the predictive value of standard tissue biomarkers remains extremely limited. Despite widespread use in other tumour types, most IHC markers provide little guidance for selecting systemic therapies. This section reviews the routine histological features that have been explored—and why none has yet proven clinically actionable as a predictor of treatment response.

### 4.1 Simple Immune Biomarkers

While PD‐L1 expression is a validated biomarker in several cancers, its performance in HCC has consistently been disappointing. Across CheckMate 040 and CheckMate 459 trials, no correlation between PD‐L1 expression and objective response or survival has been done [45]. Similarly, in Imbrave150 and Himalaya studies, PD‐L1 status did not identify responders or long‐term survivors [6, 29]. In KEYNOTE‐224, higher CPS was associated with increased response rates to pembrolizumab, but this signal was not reproduced in phase III settings and remains exploratory [46].

Due to heterogeneity, low reproducibility and lack of validated cut‐offs, PD‐L1 cannot be considered a clinically useful biomarker in HCC, despite being easily measurable.

Mismatch repair deficient (dMMR) and microsatellite instable status (MSI) are strong predictors of ICI benefit in several solid tumours. However, dMMR/MSI is exceptionally rare in HCC.

A detailed analysis in *JGH Open* demonstrated that MSI‐H is found in < 3% of advanced HCC tumours and is often associated with an atypical immune microenvironment [47]. While these rare patients may benefit from PD‐1 blockade, the prevalence is too low to justify using MSI/dMMR as a routine predictive biomarker in HCC.

Tumour mutational burden (TMB) has been explored as a predictive biomarker for ICI across several tumour types [48]. In HCC, however, its relevance appears limited [49]. HCC typically displays a relatively low mutational burden compared with highly immunogenic cancers such as melanoma or lung cancer, and available studies have not demonstrated a consistent association between TMB and response to immunotherapy [50]. Consequently, despite its theoretical rationale, TMB has not emerged as a reliable predictive biomarker in advanced HCC and is not currently used to guide treatment selection.

Glypican‐3 (GPC3), a membrane‐bound heparan sulphate proteoglycan frequently overexpressed in HCC, has also been explored as a potential biomarker in the context of immunotherapy. Beyond its diagnostic utility, recent studies suggest that GPC3 expression may be associated with tumour immune microenvironment characteristics and clinical outcomes in patients receiving immune checkpoint inhibitors [51]. However, available data remain limited and heterogeneous, and the predictive value of GPC3 expression for immunotherapy response in advanced HCC has not yet been prospectively validated.

In addition, the presence of tertiary lymphoid structures (TLS), organised aggregates of B cells, T cells and dendritic cells resembling secondary lymphoid organs, has emerged as another feature of an active tumour immune microenvironment. TLS have been associated with enhanced antitumour immune responses and improved outcomes under immune checkpoint blockade in several cancers, including HCC, further supporting the importance of immune spatial organisation in shaping immunotherapy response [52].

### 4.2 β‐Catenin (Wnt Pathway) Immunostaining

The Wnt/β‐catenin pathway is one of the most frequently altered oncogenic programmes in HCC, with activating mutations *in CTNNB1* gene (which codes for β‐catenin) found in approximately 30%–40% of cases. Alterations in other regulators of the pathway, including AXIN1, AXIN2 and APC, are also observed, although at lower frequencies, further contributing to overactivation of the Wnt signalling pathway in HCC. These alterations predominantly consist of missense mutations affecting exon 3, disrupting β‐catenin phosphorylation and degradation and resulting in constitutive pathway activation [53]. These tumours for 2/3 of them display a non‐inflamed, immune‐excluded phenotype, with reduced intratumoral T‐cell infiltration [54, 55]. Mechanistic studies demonstrate that β‐catenin pathway activation drives primary resistance to ICI by impairing dendritic‐cell recruitment and subsequent CD8^+^ T‐cell priming. This immune escape is mediated, at least in part, by reduced chemokine production (e.g., CCL5 and CCL20), as shown in preclinical models and CRISPR‐engineered HCC systems [56, 57, 58, 59]. Consistent with these observations, recent single‐cell RNA sequencing analyses have shown that tumours responding to atezolizumab–bevacizumab exhibit reduced expression of Wnt/β‐catenin–related genes, including GLUL, AXIN2 and DKK4, further supporting the association between β‐catenin signalling, immune exclusion and resistance to immunotherapy [60]. Despite its strong biological relevance, β‐catenin assessment is not clinically actionable: Routine immunohistochemistry assesses β‐catenin protein expression and localisation, but fails to reliably capture functional activation of the pathway; no standardised scoring exists, and mutational testing lacks prospective validation for treatment selection.

Given the central role of Wnt/β‐catenin signalling in HCC tumourigenesis, immune exclusion and resistance to immunotherapy, there is considerable interest in therapeutically targeting this pathway. Pharmacological inhibition of the β‐catenin transcriptional complex has shown promising activity in preclinical models of HCC. One such agent, E7386, is a selective inhibitor of the interaction between β‐catenin and its co‐activator CREB‐binding protein (CBP), effectively disrupting downstream Wnt signalling and reducing oncogenic transcriptional programmes. In combination with the tyrosine kinase inhibitor lenvatinib, E7386 exhibited synergistic antitumour effects, enhancing tumour shrinkage and survival compared with monotherapy in preclinical HCC models. This combination also activated integrated stress response pathways (e.g., ATF4), supporting a mechanistic basis for enhanced efficacy (E7386 + lenvatinib preclinical) [61].

Importantly, early clinical observations from a Phase 1b study (NCT04008797) suggest that the E7386–lenvatinib combination may have activity in patients with advanced HCC. In the dose‐escalation portion of this study, an objective response rate of approximately 36% per mRECIST was reported among evaluable patients treated with the combination, providing an initial signal of clinical efficacy (E7386 + lenvatinib Phase 1b HCC). These data, while preliminary, indicate that inhibiting the β‐catenin/CBP interaction may enhance sensitivity to systemic therapy—particularly when combined with anti‐angiogenic kinase inhibition. More recently, experimental studies have suggested that precise targeting of β‐catenin signalling may also reprogramme the tumour immune microenvironment and enhance sensitivity to immune checkpoint blockade, providing a rationale for combined therapeutic strategies [62].

Collectively, these findings reinforce the pathogenic importance of the β‐catenin axis in HCC progression and therapeutic resistance, and underscore the translational potential of Wnt/β‐catenin inhibitors as novel adjuncts to existing systemic regimens. However, larger clinical trials with robust biomarker frameworks and careful patient stratification will be required to define the true predictive value of this strategy.

### 4.3 VETC Positive Tumours

Among routine histological features, vessels encapsulating tumour clusters (VETC) represent a distinctive angiogenic vascular pattern associated with an angiogenesis‐driven tumour phenotype in hepatocellular carcinoma. VETC‐positive tumours display a strong angiogenic signature and a relatively immunosuppressed microenvironment, which has been associated with reduced sensitivity to immune checkpoint inhibition alone and can be found in 30%–40% of resected HCC. In contrast, several retrospective studies have shown that VETC positivity is associated with improved outcomes under anti‐angiogenic therapies, including tyrosine kinase inhibitors such as sorafenib and lenvatinib [63, 64]. Recently, a large multicentre study focusing on unresectable HCC confirmed that VETC‐positive tumours derive a significant survival benefit from anti‐angiogenic therapy including A + B, supporting the role of VETC as a morphologic surrogate of angiogenesis‐driven disease [65]. As VETC status can be assessed on routine liver biopsy using CD34 immunohistochemistry, it represents a simple and accessible histological biomarker candidate, although prospective validation and standardised scoring criteria are still required before clinical implementation.

## 5 Exploratory Biomarkers Awaiting Clinical Validation

Beyond clinically accessible biomarkers, an expanding landscape of exploratory tools is being investigated to better capture the biological determinants of treatment response in advanced HCC. These emerging biomarkers—derived from circulating analytes, tumour tissue or advanced computational analysis—offer valuable insights into tumour heterogeneity, immunobiology and mechanisms of treatment sensitivity. However, despite compelling early signals, none has yet met the analytical robustness, reproducibility or prospective validation required for routine clinical use. This section outlines the most promising categories of exploratory biomarkers and examines their potential, limitations and relevance for future personalised therapeutic strategies.

### 5.1 Liquid Biopsy–Derived Biomarkers

Liquid biopsy has emerged as an appealing complement to tissue‐based biomarker strategies in advanced HCC, offering a minimally invasive means to overcome sampling bias and to monitor tumour evolution in real time [66]. Circulating analytes—including cytokines, extracellular vesicles (EVs), CTCs, ctDNA and extracellular RNAs—provide dynamic insight into mechanisms of response and resistance under systemic therapy. Although these biomarkers offer clear conceptual advantages and have generated encouraging exploratory data, none has yet demonstrated the analytical reliability, standardisation or prospective validation required for clinical implementation.

#### 5.1.1 Soluble Immune Mediators: Cytokines and Extracellular Vesicle (EV)‐Derived Molecules

Soluble mediators circulating in plasma reflect systemic inflammation, immune activation and angiogenic signalling, all central to response or resistance to immunotherapy and anti‐angiogenic therapy. Several cytokines—particularly IL‐6, IL‐8 and TNF‐α—as well as angiogenic factors such as VEGF, Ang‐2 and HGF, have been associated with outcomes in exploratory cohorts. More recently, chemokines involved in T‐cell recruitment, including CXCL9, CXCL10 and CXCL11—ligands of the CXCR3 receptor, a key chemokine receptor expressed on activated immune cells—have also emerged as potential correlates of treatment response. In particular, reduced expression of CXCL9–11 and CCL5 has been associated with non‐inflamed tumour classes and poorer immune infiltration, suggesting that defective chemokine signalling may contribute to resistance to immune‐based therapies in HCC [55].

Among circulating inflammatory mediators, IL‐6 has emerged as one of the most consistently reported candidates. In a retrospective multicenter cohort of patients treated with A + B, elevated baseline IL‐6 levels were associated with reduced clinical benefit, shorter PFS and OS, and a lower probability of durable disease control [67]. Additional exploratory studies have identified other circulating inflammatory or angiogenic mediators associated with treatment outcomes, including IL‐8 and angiogenic factors such as Ang‐2 and HGF, further supporting the role of systemic inflammatory and vascular signalling in shaping therapeutic response [68, 69].

Beyond soluble mediators, tumour‐derived extracellular vesicles (EVs) have emerged as potent regulators of immune escape in HCC [69, 70]. EVs mediate intercellular communication within the tumour microenvironment and beyond, transferring small particles that can profoundly alter immune cell function. Experimental models have shown that oncogenic β‐catenin signalling reshapes the immune landscape partly through EV‐ mechanisms associated with impaired immune cell infiltration and reduced anti‐tumour immune activation [59, 71]. Beyond β‐catenin–driven contexts, several EV‐associated proteins have been implicated in suppressing anti‐tumour immunity. For instance, HCC‐derived EVs carrying 14–3‐3ζ have been shown to impair the effector function of tumour‐infiltrating T lymphocytes [72], while EV‐associated LOXL4 promotes macrophage‐mediated immune evasion by inducing PD‐L1 expression via STAT1 activation [70].

In addition to mechanistic studies, emerging translational investigations have evaluated circulating EVs as potential clinical biomarkers. For example, EV‐associated protein signatures detected in patient plasma using multiplex cytometry platforms such as MACSPlex‐EV have shown promise for the diagnosis and recurrence detection of HCC, highlighting their potential clinical applicability. Proteomic analyses of EVs derived from patient‐derived xenograft models have further demonstrated that tumour‐derived exosomal cargo can reflect tumour identity and molecular characteristics, suggesting that EV proteomics may provide a minimally invasive window into tumour biology.

Overall, soluble biomarkers capture relevant biological pathways but suffer from poor specificity, high inter‐patient variability, lack of standardised assays and susceptibility to confounding by other pathological contexts, like cirrhosis and systemic inflammation. Consequently, despite strong biological rationale, these circulating mediators currently remain exploratory biomarkers and have not yet demonstrated sufficient robustness or prospective validation to guide treatment selection in clinical practice.

### 5.2 Circulating Tumour Cells (CTCs) and Circulating Immune Cells

CTCs provide a unique opportunity to examine viable tumour cells shed into the bloodstream, offering integrative information on genomic alterations, transcriptomic programmes, protein expression and metastatic potential [66, 73, 74, 75]. Importantly, circulating tumour cells may originate from both the primary tumour and metastatic lesions, thereby providing a global, systemic snapshot of tumour biology rather than a site‐specific view. Several studies have explored PD‐L1 expression on CTCs as a biomarker for ICI response, reporting associations between high PD‐L1^+^ CTC burden and improved PFS and ORR in patients treated with anti‐PD‐1 regimens [76].

Beyond tumour cells, circulating immune subsets—such as exhausted T cells, myeloid‐derived suppressor cells or specific NK cell phenotypes—have been proposed as potential mediators of differential response to immunotherapy [74, 77, 78, 79]. Although these signatures provide mechanistic insight, their clinical applicability remains limited by technical challenges, analysis platform heterogeneity and the need for fresh samples and high‐parameter flow cytometry or single‐cell technologies.

Overall, both CTCs and circulating immune profiles remain high‐value mechanistic biomarkers, but their lack of sensitivity and reproducibility currently prevent any clinical implementation.

### 5.3 Circulating Tumour DNA (ctDNA), Methylated DNA Signatures and Circulating miRNAs

Among liquid biopsy analytes, ctDNA has generated the greatest enthusiasm due to its potential to capture spatial tumour heterogeneity, track clonal evolution and provide real‐time molecular correlates of treatment response [80].

In HCC, higher baseline ctDNA levels correlate with increased tumour burden, vascular invasion and inferior survival outcomes [73]. The most compelling evidence for ctDNA as a dynamic biomarker in advanced HCC comes from exploratory analyses of A + B. In an analysis of patients enrolled in the GO30140 trial, personalised ctDNA assays (Signatera, tumour‐informed sequencing) were successfully designed for 47 of 48 patients, with ctDNA detected in 96% at baseline [81]. Higher baseline ctDNA levels correlated with larger tumour burden, reinforcing its biological consistency. Most importantly, longitudinal ctDNA clearance strongly mirrored radiological benefit: at cycle 4, ctDNA became undetectable in 70% of complete responders, 27% of partial responders, 9% of patients with stable disease, and in none with radiological progression. Patients achieving early ctDNA clearance experienced markedly prolonged progression‐free survival, whereas persistent ctDNA positivity predicted early resistance. These findings provide proof‐of‐concept that ctDNA dynamics may function as an early, treatment‐specific biomarker of response under A + B.

Beyond mutation profiling, epigenetic approaches—particularly DNA methylation analysis—have emerged as a promising strategy for the identification of novel biomarkers with potential clinical utility in cancer management. Regarding liquid biopsy approaches, methylated cell‐free DNA represents a sensitive tool for detecting minimal residual disease and tumour burden [82]. Although most studies have focused on early tumour detection, the same technology could theoretically predict treatment sensitivity by quantifying tumour‐derived methylation signatures before and during treatment course. However, no methylation panel has yet been validated for treatment prediction in advanced HCC [83].

Circulating microRNAs (miRNAs) are stable, non‐coding RNA molecules that reflect tumour biology and have shown prognostic value in HCC. However, their use as *predictive* biomarkers for systemic therapy response remains exploratory [84]. Multiple preclinical studies demonstrate that miRNAs modulate pathways linked to chemoresistance and immune escape—such as autophagy, apoptosis and PD‐L1 regulation—but no miRNA has yet achieved clinical validation to predict response to TKIs, cytotoxic chemotherapy or ICIs in HCC.

Despite their strong conceptual appeal and growing technical sophistication, ctDNA, cell‐free methylated DNA signatures and circulating miRNAs remain investigational due to high costs, variability in extraction and sequencing methods, lack of standardised thresholds and absence of prospective validation in therapeutic decision‐making.

### 5.4 Gut Microbiota

Increasing evidence suggests that the gut microbiota may influence response to immune checkpoint inhibitors through modulation of systemic and hepatic immune responses [85, 86, 87, 88]. In hepatocellular carcinoma, the gut–liver axis is particularly relevant given the close anatomical and immunological connection between the intestinal microbiome and hepatic immune regulation. Several studies have reported associations between specific microbiota compositions and outcomes under immunotherapy. In particular, enrichment in taxa such as *Akkermansia muciniphila* , *Ruminococcaceae* and *Faecalibacterium* has been associated with improved responses to immune checkpoint blockade, whereas dysbiosis and overrepresentation of pro‐inflammatory species may correlate with treatment resistance. However, these observations remain largely exploratory, with substantial heterogeneity in sequencing methods, patient populations and analytical pipelines. As a result, the gut microbiome has not yet emerged as a clinically actionable biomarker in advanced HCC, although it represents a promising area for future translational research.

## 6 Tissue Biomarkers

Although tissue‐based analyses have provided some of the most informative insights into tumour biology and treatment response in advanced HCC, their clinical applicability is intrinsically constrained by the limitations of tumour sampling. Percutaneous liver biopsy remains an invasive procedure in cirrhotic patients, frequently limited by coagulopathy and portal hypertension and often yields small specimens with variable tumour cellularity. In addition, tissue availability is rapidly exhausted by routine diagnostic work‐up and ancillary molecular analyses, restricting the feasibility of large‐scale or iterative profiling. These constraints strongly argue for the development of complementary, less invasive approaches capable of capturing tumour heterogeneity and dynamic treatment response.

### 6.1 Transcriptomic Immune Signatures and Molecular Correlates of Treatment Response

At the genomic level, NGS‐based tumour genotyping has clarified the landscape of recurrent mutations but has not yet yielded actionable predictive markers. Although alterations in LRP1B, associated with higher TMB, correlate with worse prognosis [89], they have not shown treatment‐specific predictive value, especially regarding immunotherapy‐based combinations. Similarly, TP53 mutations remain prognostically relevant but not predictive of ICI benefit. Prospective sequencing cohorts confirm this lack of clinically deployable genomic predictors for immunotherapy [50]. A more recent integrative analysis identified transcriptional programmes linked to response to anti‐PD‐1 therapy—particularly interferon‐related signatures, exhaustion markers and angiogenic states—but these signals remain exploratory [90].

Epigenomic biomarkers, particularly DNA methylation profiles, are increasingly investigated in HCC [82]. Methylation analysis of circulating DNA (cDNA) has shown strong potential for prognosis assessment and longitudinal monitoring, and early clinical reports suggest that cDNA methylation dynamics may help anticipate radiological and/or pathological response in patients receiving neoadjuvant and systemic therapies [91]. However, in advanced HCC, epigenomic markers remain largely exploratory as predictive tools, and most available evidence is derived from association studies, mechanistic work on drug resistance (including under TKIs) and ongoing clinical evaluation rather than prospective treatment‐stratification trials [92].

Major advances in RNA sequencing, multi‐omic profiling and single‐cell technologies have substantially improved our understanding of tumour–immune interactions in HCC, revealing transcriptomic programmes that shape sensitivity or resistance to systemic therapies. As highlighted in recent reviews on the evolving molecular classification of HCC [9, 93]; HCC can be broadly stratified into immune‐hot and immune‐cold phenotypes. Immune‐hot tumours display high cytotoxic T‐cell infiltration, active IFN‐γ signalling and enriched antigen‐presentation pathways, features consistently associated with improved response to immune checkpoint blockade. Conversely, immune‐cold tumours exhibit T‐cell exclusion, myeloid‐driven inflammation, and angiogenic signalling, forming an immunosuppressive microenvironment conducive to primary resistance.

The most comprehensive analysis to date comes from the GO30140 and IMbrave150 multi‐omic study [94]. In these trials, pre‐existing immunity—reflected by elevated CD8+ T‐cell density, high PD‐L1 (CD274) expression and a strong T‐effector signature—was associated with better outcomes under A + B. In contrast, reduced clinical benefit was linked to high Treg/Teff ratios and the expression of oncofetal genes such as GPC3 and AFP, suggesting persistent immunosuppression. Importantly, the authors showed that anti‐VEGF therapy may synergise with anti‐PD‐L1 by modulating angiogenesis, dampening Treg proliferation and reprogramming myeloid inflammation. These insights were validated not only through bulk RNA‐seq analysis but also by paired biopsies and mechanistic mouse models.

More recently, an integrative transcriptomic analysis demonstrated that HCC molecular subtypes are associated with differential clinical benefit from anti–PD‐L1 and anti‐VEGF combination therapy [69]. By analysing tumour samples from GO30140 and IMbrave150, three reproducible subtypes were identified—cholangiocyte‐like, hepatocyte‐like and progenitor‐like—each associated with distinct immune landscapes and treatment outcomes. The cholangiocyte‐like subtype exhibited the highest objective response rates and longest progression‐free survival under A + B, whereas progenitor‐like tumours, characterised by oncofetal gene expression (*GPC3*, *AFP*), high Treg/Teff ratios and an immune‐excluded phenotype, derived limited benefit. These subtype‐specific differences translated into a clear survival advantage for A + B versus sorafenib in cholangiocyte‐like and hepatocyte‐like tumours, but not in progenitor‐like disease. While biologically compelling and consistent with previously described immune and angiogenic correlates of response [94], this classification relies on RNA sequencing and lacks prospective validation, currently limiting its clinical transposability.

Building on this work too, a refined transcriptomic classifier—the A + B Response Signature (ABRS)—was developed to integrate T‐cell activation, IFN‐γ signalling, antigen presentation and VEGF‐related pathways. High ABRS expression correlates with higher response rates and longer PFS on A + B [95], although the signature remains exploratory given the need for RNA‐seq, lack of harmonisation and absence of prospective validation.

Single‐cell RNA sequencing has further dissected the tumour microenvironment with unprecedented resolution [60]. A recent study defined 21 cell‐type–specific transcriptional signatures and identified two distinct patterns of clinical benefit to atezolizumab–bevacizumab: (1) An immune‐competent phenotype characterised by abundant CD8+ Teff cells and CXCL10+ pro‐inflammatory macrophages; and (2) An angiogenesis‐driven phenotype, marked by reduced expression of the VEGF co‐receptor NRP1, predicting improved survival with A + B versus sorafenib. Resistance, conversely, was associated with TREM2+ macrophages, CD14+ monocytes and Notch pathway activation, delineating a ‘Resistant’ molecular subset (Single‐cell RNA sequencing–derived signatures study). However, despite its high cellular resolution, single‐cell RNA sequencing does not preserve spatial, organisational or conformational information within the tumour microenvironment, limiting its ability to capture cell–cell interactions, tissue architecture and functional immune niches that may critically influence therapeutic response.

Other transcriptomic frameworks have reinforced the value of molecular subtyping. A large multi‐cohort analysis integrating single‐cell data identified five transcriptomic classes with distinct immune landscapes and therapeutic sensitivities: inflamed, T‐cell–excluded, immunosuppressed, proliferative and globally resistant subtypes [96]. These classes demonstrated differential responsiveness to immunotherapy, targeted agents and loco‐regional therapies, underscoring the relevance of tumour–immune contexture in treatment selection.

Beyond bulk transcriptomic signatures, the spatial organisation of the tumour immune microenvironment is emerging as another important determinant of response to ICI. Using highly multiplexed imaging mass cytometry combined with spatial single‐cell analysis, Salie et al. [97] characterised the immune architecture of HCC and identified three major spatial immunotypes: immune‐depleted, compartmentalised and immune‐enriched tumours. These spatial patterns reflected distinct modes of immune‐cell organisation within the tumour microenvironment and were associated with differential outcomes under ICI therapy, with immune‐enriched tumours displaying significantly improved progression‐free survival. Interestingly, the presence of even a limited immune‐enriched niche appeared sufficient to drive favourable clinical outcomes.

Beyond immune cell composition, the tumour microenvironment in HCC is also shaped by non‐immune stromal components, particularly hepatic stellate cells, which play a central role in immune regulation and extracellular matrix remodelling. Activated stellate cells contribute to the recruitment and polarisation of macrophages through cytokine and chemokine signalling, thereby promoting an immunosuppressive milieu. In parallel, they drive the deposition of extracellular matrix components, increasing tissue stiffness and forming physical barriers that limit immune‐cell infiltration within the tumour. These dual immunoregulatory and structural functions position hepatic stellate cells as key modulators of tumour immune architecture and potential determinants of response to immunotherapy.

These findings further highlight the importance of the tumour immune microenvironment architecture in shaping immunotherapy response, although such spatially resolved approaches remain primarily investigational [98, 99].

Beyond transcriptomics, metabolomic–transcriptomic integration of peri‐tumoral tissue has identified additional resistance pathways [96]. Upregulation of COMMD3–BMI1 and depletion of Dephospho‐CoA were associated with nonresponse to atezolizumab, findings supported by functional studies and animal models, highlighting novel metabolic correlates of immune resistance.

Finally, computational and spatial multi‐omic approaches have begun to unravel spatial determinants of response. Spatial transcriptomics analyses from neoadjuvant trials and virtual clinical simulations highlight the importance of CD8+ T‐cell–macrophage proximity, tumour vasculature patterns and TGF‐β signalling in shaping ICI response, providing further mechanistic rationale for combined anti‐VEGF and immune checkpoint strategies [100, 101].

Taken together, transcriptomic and molecular biomarkers derived from tumour tissue provide some of the most sophisticated biological insights into mechanisms of treatment response in advanced HCC. However, despite their strong mechanistic relevance, none has yet fulfilled the key requirements for clinical implementation—namely standardisation, reproducibility, accessibility and prospective validation. Moreover, broad molecular screening strategies remain costly, often limited by tissue availability in cirrhotic patients, and rarely identify actionable alterations linked to approved targeted therapies. As a result, comprehensive genomic profiling in HCC currently contributes more to biological understanding than to treatment selection. The true value of these approaches therefore lies in informing tumour biology and guiding the development of next‐generation, integrative predictive models rather than in routine clinical decision‐making.

### 6.2 Artificial Intelligence–Based Pathology

Artificial intelligence (AI) has rapidly emerged as a powerful tool to extract quantitative, clinically meaningful information from routine histopathology. In HCC, AI‐driven pathology—often referred to as *pathomics*—can capture complex morphological and spatial features that are imperceptible to human observers, offering a new dimension of tumour profiling beyond genomics and transcriptomics [102].

The most compelling evidence for AI as a predictive biomarker to date comes from a multicenter retrospective study published in Lancet Oncology 2023, where deep‐learning models applied to standard H&E slides identified morphological patterns associated with differential sensitivity to A + B [103]. This work showed that computational pathology could stratify patients according to expected benefit from immunotherapy–anti‐VEGF combinations, using only baseline tissue—highlighting its remarkable scalability and clinical appeal.

Earlier foundational studies, particularly those by Calderaro et al. [104] demonstrated that AI can accurately classify HCC subtypes, infer molecular and immune features and identify prognostic patterns from routine histology. These efforts established the proof‐of‐concept that digital pathology can serve as a surrogate for more complex molecular assays, providing a ready‐to‐use substrate for predictive modelling in HCC.

Beyond prediction of treatment response, several recent studies have developed AI‐driven pathomic prognostic models integrating morphological signatures with clinical parameters to predict recurrence, tumour aggressiveness and microenvironmental features [102, 105]. More recently, AI‐based analysis of routine histology has also been shown to predict outcome‐associated molecular profiles and vascular invasion in HCC, further illustrating the ability of computational pathology to infer biologically relevant tumour characteristics directly from tissue morphology [106].

These models underscore the ability of AI to capture microarchitectural patterns reflecting stromal remodelling, immune exclusion and tumour differentiation—dimensions highly relevant to systemic therapy response.

However, despite this strong conceptual framework and growing methodological sophistication, AI‐derived biomarkers remain far from clinical implementation. Key limitations include:

- Reliance on proprietary algorithms and non‐standardised pipelines,
- Limited reproducibility across staining, scanning and image‐processing platforms,
- Lack of prospective, external validation,
- Absence of regulatory frameworks for diagnostic deployment,
- And the challenge of ensuring interpretability for clinical decision‐making.

Consequently, while AI‐based pathology represents one of the most promising avenues for future predictive biomarker development in HCC—capable of analysing routine biopsy material at unparalleled scale—its use remains strictly exploratory. No AI‐driven signature has yet achieved the level of validation required to guide therapeutic selection in advanced HCC.

### 6.3 Current Status and Outlook for Proteomics

Proteomics has emerged as a particularly relevant exploratory approach to address the limitations of genomic and transcriptomic biomarkers in advanced HCC [107, 108, 109, 110]. While DNA and RNA profiling inform on potential regulatory programmes, the proteome represents the functional endpoint of biological regulation, integrating epigenetic, transcriptional, post‐transcriptional and post‐translational mechanisms [111, 112, 113]. As direct effectors of cellular behaviour, proteins govern key oncogenic processes—including metabolism, angiogenesis, immune modulation and therapeutic resistance—making proteomic profiling a more proximal readout of tumour biology and treatment sensitivity than upstream molecular layers [110].

Proteomic analyses are primarily performed using mass spectrometry–based technologies [111, 114]. Targeted approaches (such as SRM/MRM or PRM) enable accurate quantification of predefined proteins of interest but are inherently hypothesis‐driven and poorly suited to unbiased exploration of complex biological networks. In contrast, non‐targeted, discovery‐based proteomics allows global interrogation of the proteome without prior assumptions, and is therefore particularly adapted to heterogeneous diseases such as HCC. Among available technologies, high‐resolution liquid chromatography–tandem mass spectrometry (LC–MS/MS) has become the reference method, enabling the identification and relative quantification of several thousand proteins from complex clinical samples, including formalin‐fixed paraffin‐embedded tissue, after enzymatic digestion and chromatographic separation [115, 116].

Beyond its technical performance, proteomics captures layers of biological regulation that remain largely inaccessible to genomic or transcriptomic analyses, such as post‐translational modifications, protein–protein interactions, pathway activation states and fine metabolic adaptations. Consistently, multi‐omic studies have demonstrated only a modest correlation between transcript abundance and protein expression, underscoring the need to directly interrogate the proteome when aiming to identify functional biomarkers.

Although clinical applications remain limited, proteomics has already demonstrated tangible translational value in liver disease. High‐resolution tissue proteomic profiling enabled the identification of argininosuccinate synthase 1 (ASS1) as a specific marker of the sonic hedgehog hepatocellular adenoma subtype, subsequently translated into routine diagnostic practice following immunohistochemical validation [19, 117, 118].

This example illustrates the ability of proteomics to uncover biologically meaningful tissue signatures that are not readily detectable using conventional molecular approaches.

Building on these foundations, the laboratory has developed a tissue‐based proteomic strategy combining unbiased high‐resolution profiling with machine‐learning–driven ‘profile matching’. This approach relies on the generation of global proteomic profiles from routine FFPE biopsies, the construction of reference protein expression signatures associated with defined biological or clinical phenotypes, and the classification of individual samples through comparison with these reference profiles. By embracing biological complexity through multi‐protein signatures rather than single markers, and by remaining compatible with routine diagnostic material, this strategy offers a realistic path toward translational application [119].

To date, no proteomic signature predicting response to systemic therapy in advanced HCC has been validated at scale, representing a major unmet clinical need. In a context where genomic and transcriptomic biomarkers have shown limited ability to stratify treatment response, proteomics may represent a key opportunity to identify functionally grounded predictive tools and to support the development of biologically informed precision medicine in advanced HCC.

## 7 Radiomics

Radiomics has rapidly evolved as a non‐invasive imaging‐based biomarker strategy capable of capturing tumour and microenvironmental heterogeneity beyond what is appreciable by visual assessment. By extracting high‐dimensional quantitative features from standard Computed Tomography (CT) or Magnetic Resonance Imaging (MRI), and increasingly integrating deep learning, radiomics offers a scalable framework to characterise tumour biology, immune contexture and potential treatment responsiveness. In advanced HCC—where systemic therapy decisions rely heavily on imaging—radiomics and radio‐immunomics represent particularly appealing exploratory tools, evaluated mainly in retrospective imaging cohorts, though none has yet reached the level of validation required for clinical adoption [120].

Recent pilot studies support the feasibility of using pre‐treatment CT‐based radiomics to predict ICI efficacy:

In a cohort of 55 patients treated with immunotherapy, an AI‐driven model integrating radiomic features from the whole liver and viable tumour regions achieved accuracies up to 86% for distinguishing responders from non‐responders by mRECIST, with particularly high specificity when combining liver and tumour features [121].

Similarly, another study analysing 54 patients identified radiomic signatures—particularly GLRLM and NGTDM texture metrics, shape descriptors and adjacent‐tissue intensity features—that significantly differed between responders and non‐responders, outperforming several machine‐learning approaches and validating key feature combinations in an external cohort [122].

Extending these findings, a large two‐centre analysis of 152 patients treated with A + B demonstrated that integrated radiomic–clinical models markedly outperformed established clinical tools such as BCLC stage and ALBI grade. The integrated model achieved AUCs of 0.89 (derivation) and 0.75 (validation), and radiomic‐defined high‐risk groups had significantly shorter OS and PFS as well as lower ICI response rates. Radiomic risk emerged as the strongest independent predictor of survival [123].

Together, these exploratory biomarker strategies—ranging from liquid biopsy analytes to tissue‐based molecular profiling, AI‐assisted pathology and radiomics—highlight remarkable efforts of emerging tools aimed at refining treatment prediction in advanced HCC. Each offers unique biological or functional insights, yet all share a common limitation: none has yet demonstrated the analytical robustness, reproducibility or prospective validation required for routine clinical use. Their collective promise lies not in isolated markers, but in the possibility of integrated, multi‐modal approaches capable of capturing the full complexity of tumour biology and host–liver interactions. As these technologies mature, they are poised to complement, rather than replace, established clinical biomarkers—paving the way toward more precise and personalised systemic therapy strategies.

**FIGURE 1:**
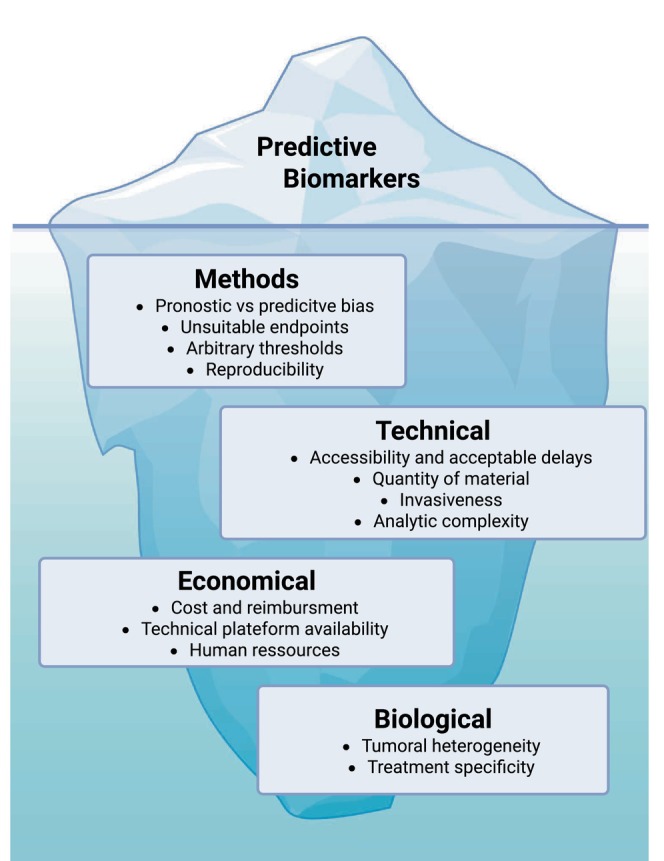
Conceptual framework of biomarkers associated with response to systemic therapies in advanced hepatocellular carcinoma. Biomarkers are organised as concentric layers reflecting a continuum of clinical transposability and level of supporting evidence, rather than discrete categories. The central blue circle represents clinically integrated or actionable biomarkers, primarily dynamic or on‐treatment markers that can inform real‐time therapeutic decision‐making, such as AFP kinetics. The intermediate green layer includes biologically relevant biomarkers supported by emerging clinical evidence but not yet validated for routine use. These encompass tumour‐intrinsic and microenvironment‐related features derived from multi‐omics approaches (e.g., proteomics, transcriptomic signatures, NGS genotyping), as well as specific pathways and phenotypes such as β‐catenin activation, VETC pattern and anti‐drug antibodies. The outer beige layer comprises exploratory biomarkers that remain investigational due to methodological heterogeneity, lack of standardisation or insufficient prospective validation. These include circulating biomarkers (ctDNA, exosomes, circulating miRNAs, cytokines), microbiome‐related signatures, AI‐based pathology, radiomics and liver disease aetiology. This framework highlights the progressive transition from exploratory biological signals to clinically actionable tools, reflecting the current gap between biomarker discovery and implementation in precision medicine for advanced HCC.

Given the heterogeneity of reported biomarkers and their treatment‐specific implications, Table 1 and Figure 1 provide an overview and a comparative synthesis of biomarkers associated with response to anti‐angiogenic therapies versus immunotherapy‐based combinations.

**TABLE 1:** Differential biomarkers of response according to systemic treatment class in advanced hepatocellular carcinoma.

Biomarker category	Anti‐angiogenic therapies	Immunotherapy‐based combinations	Biomarker timing	Evidence level	External validation	Assay feasibility	Clinical readiness
Clinical	ALBI score (prognostic conditioning of benefit)	ALBI score (treatment tolerance, access to benefit)	Baseline	Prospective cohorts/Post hoc trials	Yes	Routine	Prognostic
Circulating	AFP kinetics; Ang‐2, HGF; Inflammation indices	Anti‐drug antibodies; AFP kinetics; CRAFITY score; IL‐6	Baseline/On‐treatment	Retrospective cohorts/Post hoc analyses	Partial	Routine/ELISA	Monitoring/Exploratory
Routine pathology	VETC positivity (CD34 vascular pattern)	PD‐L1 IHC; β‐catenin IHC; dMMR/MSI	Baseline	Retrospective cohorts/Translational studies	Limited	Routine IHC	Investigational
Imaging (standard)	RECIST 1.1; mRECIST response	RECIST 1.1; mRECIST response	On‐treatment	Prospective trials	Yes	Routine	Standard monitoring
Exploratory	Transcriptomic angiogenic signatures; Radiomics (vascular features); Proteomic angiogenesis pathways	Transcriptomic immune signatures (ABRS); ctDNA clearance; AI‐pathology; Proteomics	Baseline/On‐treatment	Translational cohorts	Limited	Complex	Exploratory

Abbreviations: AFP, alpha‐fetoprotein; ALBI, albumin–bilirubin score; Ang‐2, angiopoietin‐2; CRAFITY, C‐reactive protein and AFP; ctDNA, circulating tumour DNA; dMMR, deficient mismatch repair; HGF, hepatocyte growth factor; IL‐6, interleukin‐6; mRECIST, Modified Response Evaluation Criteria in Solid Tumours; MSI, microsatellite instability; PD‐L1, programmed death‐ligand 1; RECIST 1.1, Response Evaluation Criteria in Solid Tumours version 1.1; VETC, vessels encapsulating tumour clusters.

## 8 Conclusion and Future Directions

Despite the rapid expansion of systemic therapies for advanced HCC, no biomarker has yet achieved sufficient robustness to guide first‐line treatment selection. Most clinically accessible tools remain predominantly prognostic, informing survival or treatment tolerance rather than identifying which patients will benefit preferentially from immunotherapy‐based combinations or anti‐angiogenic therapies. Among circulating markers, anti‐drug antibodies represent one of the few treatment‐specific signals with potential clinical relevance, while dynamic biomarkers such as early AFP decline or ctDNA clearance appear more closely associated with treatment activity but remain insufficiently standardised for routine implementation.

**FIGURE 2:**
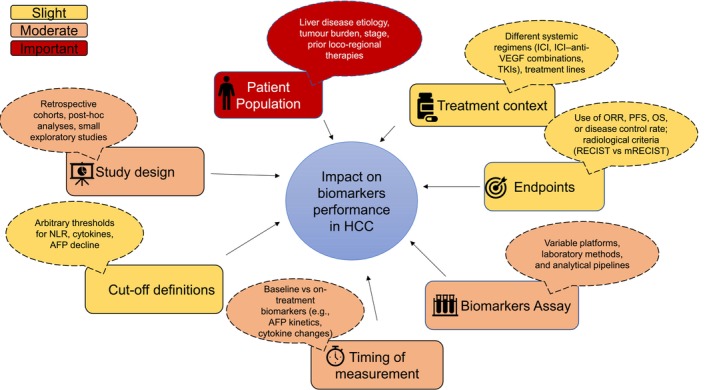
Major sources of heterogeneity in studies evaluating biomarkers of treatment response in advanced HCC.

Exploratory biomarkers derived from liquid biopsy, tumour tissue profiling, radiomics and AI‐driven pathology have substantially advanced biological understanding of response and resistance mechanisms. However, their translation into clinical practice remains limited, not only by biological complexity and tumour heterogeneity, but also by practical constraints inherent to routine care. Practical constraints—including assay availability, cost, reimbursement, turnaround time, tissue requirements and the need for specialised technical expertise—substantially limit large‐scale clinical implementation. In addition, the frequent reliance on arbitrary thresholds and the limited treatment specificity of many biomarkers further weaken their clinical applicability (Figure 2).

Methodological challenges also persist. Endpoint selection critically shapes interpretation. In advanced HCC, OS is heavily influenced by liver dysfunction, comorbidities, treatment tolerance and competing risks of death, often obscuring true drug sensitivity which complicates cross‐study comparisons and the interpretation of real‐world survival data. In contrast, response‐based endpoints provide a more direct, albeit still imperfect, assessment of treatment activity. Most biomarker candidates described to date derive from retrospective or post hoc analyses, and therefore should be interpreted as associative signals rather than causal predictors of treatment benefit.

**FIGURE 3:**
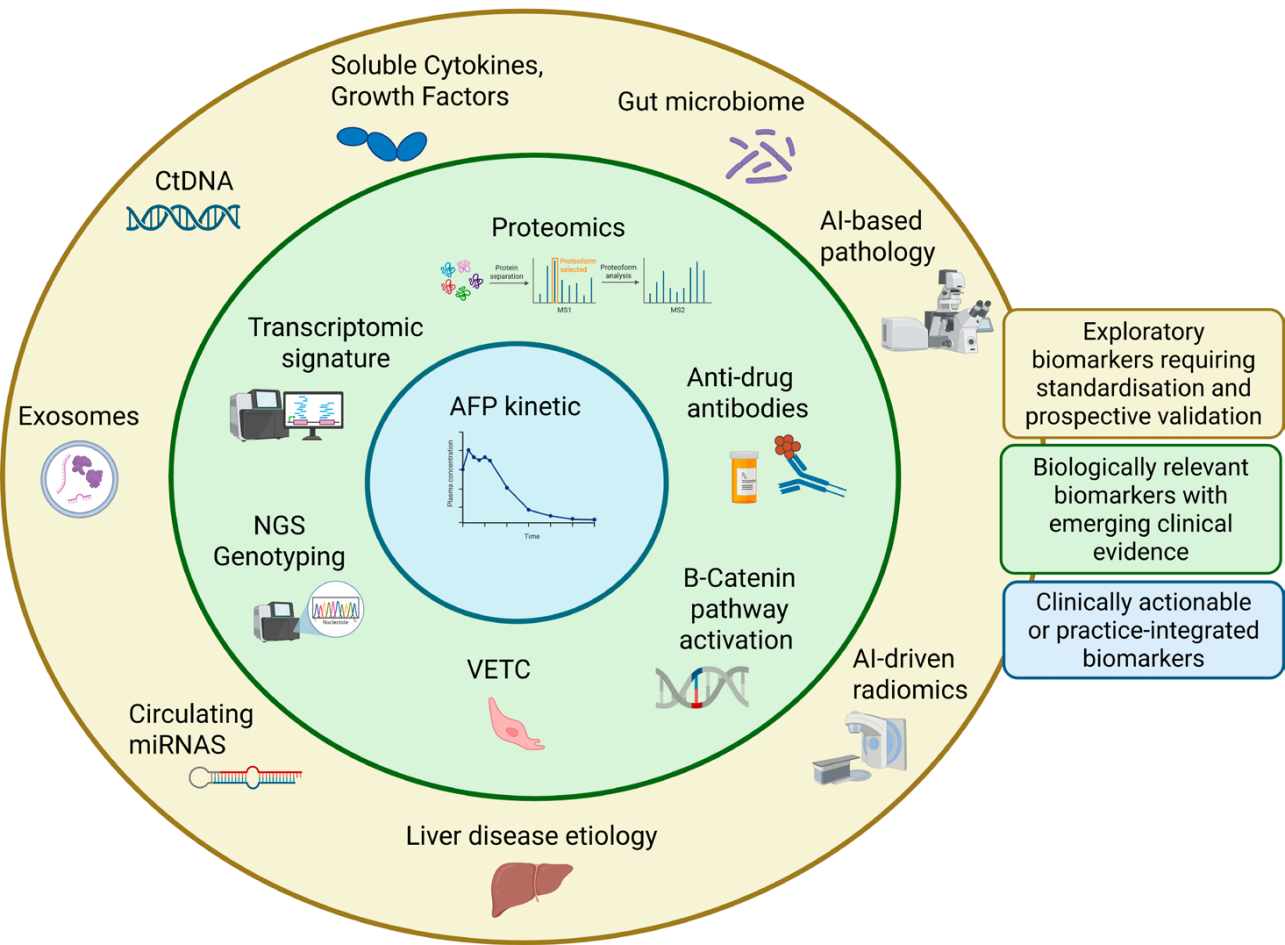
Why biomarkers fail?

The heterogeneity of results observed across biomarker studies in HCC is largely explained by differences in patient populations, treatment contexts, study endpoints, biomarker assays and cut‐off definitions. These methodological variations complicate cross‐study comparisons and partly explain why many promising biomarkers have failed to achieve external validation (Figure 3).

Within this landscape, proteomics represents a particularly promising avenue. By capturing the functional output of tumour biology, proteomic profiling may help identify pathway‐level determinants of treatment sensitivity that are not accessible through upstream genomic or transcriptomic analyses. When integrated with machine‐learning approaches, proteomics has the potential to improve biological interpretability while moving closer to clinically actionable prediction.

Ultimately, progress toward biomarker‐driven precision medicine in advanced HCC will likely rely on integrated, multi‐modal strategies combining tissue‐ and blood‐based biomarkers, dynamic functional readouts and advanced imaging within prospective validation frameworks. Bridging the gap between biological insight and real‐world feasibility remains the central challenge for the next generation of biomarker research in advanced HCC.

## Author Contributions

M.D. conceptualised the study, performed the literature review and drafted the manuscript. J.‐F.B. supervised the work and critically revised the manuscript. S.A. and G.M. contributed to data interpretation and manuscript revision. All authors approved the final version of the manuscript.

## Funding

The authors have nothing to report.

## Conflicts of Interest

M.D.: Roche, Servier, AZ. J.‐F.B.: Bayer, ESAI, IPSEN, ROCHE, ASTRA‐ZENECA, BMS.

## Data Availability

The data that support the findings of this study are available from the corresponding author upon reasonable request.
